# The impact of artificial intelligence literacy on learning engagement among university students: the mediating role of psychological capital and the moderating role of professional identity

**DOI:** 10.3389/fpsyg.2026.1892204

**Published:** 2026-09-11

**Authors:** Ning Wang

**Affiliations:** School of Tourism Management, Henan Finance University, Zhengzhou, China

**Keywords:** artificial intelligence literacy, learning engagement, professional commitment, psychological capital, undergraduate students

## Abstract

The rapid proliferation of artificial intelligence (AI) in higher education has positioned AI literacy as an essential student competency, yet the mechanistic pathways linking this competency to academic outcomes remain insufficiently understood. This study investigates the direct and indirect effects of AI literacy on learning engagement, with a specific focus on the mediating role of psychological capital (PsyCap) and the moderating role of career commitment.Employing a stratified random sampling design, we conducted a cross-sectional questionnaire survey among 1,198 undergraduate students across four universities in Zhengzhou, Henan Province. Path analysis was performed using partial least squares structural equation modeling (PLS‑SEM), and bootstrap resampling was applied to test both mediation and moderated mediation effects.The results reveal that AI literacy exerts a direct, positive influence on learning engagement. Notably, psychological capital partially mediates this relationship, indicating that AI literacy not only directly drives engagement but also indirectly facilitates it by enhancing students’ self‑efficacy, hope, optimism, and resilience. Furthermore, career commitment moderates the effect of psychological capital on learning engagement: the indirect effect of AI literacy through psychological capital becomes stronger as students’ career commitment intensifies.Collectively, these findings delineate a resource‑transformation pathway—from AI literacy to psychological capital to learning engagement—and identify career commitment as a critical boundary condition. The results carry theoretical implications for understanding the interplay between digital competencies and positive psychological resources, and offer practical guidance for designing educational interventions that synergistically integrate AI training, psychological capital cultivation, and professional identity reinforcement in the era of AI‑enhanced education.

## 1 Introduction

The rapid proliferation and widespread adoption of generative artificial intelligence (GenAI) technologies have profoundly transformed the daily learning practices of university students. Research indicates that since the release of ChatGPT in late 2022, over 60% of Chinese university students have used generative AI technologies during the 2023–2024 academic year, with academic use occurring more frequently than non-academic use (Cao et al., 2025). Xiao et al. (2025) further discovered based on a survey of 486 Chinese college students that over 70% of the students actively use generative AI. However, large-scale survey evidence suggests that while the use of generative AI in academic tasks is positively associated with cognitive and emotional engagement, it may also reduce students’ active learning behaviors and learning motivation, with this relationship being significantly moderated by the learning context (Guo X. et al., 2025; Guo F. et al., 2025). These findings suggest that reliance solely on usage behavior or frequency as proxy indicators is insufficient to explain individual differences in how AI affects learning. In effect, students’ effective utilization of AI technologies depends not merely on whether or how often they use them, but rather on their capacity to comprehend, evaluate, and critically engage with AI—that is, their AI literacy (Carl et al., 2022; Kit et al., 2021; Laupichler et al., 2023). Yan et al. (2025) demonstrated through a 16‑week process‑oriented intervention that university students progressively internalize GenAI knowledge, develop sophisticated prompt engineering skills grounded in metacognitive awareness, and shift their perception of GenAI from a mere tool to a context‑sensitive partner requiring both exploration and caution. As a key predictor of students’ academic achievement and an indicator of higher education quality (Schaufeli et al., 2002), learning engagement—a positive psychological state characterized by vigor, dedication, and absorption—has long been a central concern in educational psychology. A substantial body of empirical research has established that the degree of student engagement in the learning process is directly associated with critical thinking, problem-solving abilities, creativity, and ultimate learning outcomes. Therefore, in the new era in which AI technologies are deeply embedded in learning environments, exploring how AI literacy influences learning engagement and its underlying mechanisms is not merely a natural extension of academic inquiry but also an imperative response to the demands of the times.

Accumulating evidence suggests that AI literacy significantly influences learning engagement (Kit et al., 2021; He et al., 2025; Yin et al., 2025), enhances students’ academic performance and self-efficacy (Bećirović et al., 2025), and facilitates effective collaboration with intelligent technologies (Pramod, 2026). Despite the growing body of research on AI literacy and its educational implications, three critical theoretical gaps remain unaddressed. First, existing studies have predominantly examined the direct relationship between AI literacy and learning engagement (Guo X. et al., 2025; Guo F. et al., 2025; Yin et al., 2025), whereas the underlying psychological mechanisms through which AI literacy translates into sustained learning behaviors have received scant empirical attention. Second, although psychological capital (PsyCap) has been consistently identified as a robust predictor of learning engagement (Siu et al., 2014; Martínez et al., 2019; Fu and Qiu, 2023), whether and how it serves as a mediating pathway between AI literacy and learning engagement has not been directly tested. Third, the boundary conditions that may amplify or weaken the effectiveness of psychological capital in promoting learning engagement remain largely unexplored, particularly regarding the role of students’ identification with their academic major. In the era of AI, these issues are critical not only for whether individuals can achieve effective learning but also for whether higher education can translate technological dividends into genuine talent cultivation quality.

To address these gaps, this study proposes a moderated mediation model that, for the first time, integrates AI literacy, psychological capital, and professional commitment into a unified theoretical framework. This framework is anchored in the Conservation of Resources Theory (COR theory), which serves as the core theoretical lens, while also drawing upon Social Cognitive Theory, Broaden-and-Build Theory, Self-Determination Theory, Social Identity Theory, and Self-Verification Theory as complementary perspectives to provide a multitheoretical explanatory architecture. This model advances the literature in three key ways. First, drawing upon Conservation of Resources Theory and Broaden-and-Build Theory, the study posits a “resource gain spiral” pathway wherein AI literacy, as a technological cognitive resource, accumulates into psychological capital and subsequently enhances learning engagement—a chain-of-effects mechanism that moves beyond the simplistic direct-effect approaches of prior work. Second, informed by Social Identity Theory and Self-Verification Theory, the study identifies professional commitment as a critical boundary condition that activates the translation of psychological resources into behavioral engagement, thereby repositioning professional commitment from a mere direct predictor to a contextual amplifier. Third, by empirically validating this integrated framework, this study bridges the theory–practice gap in AI-enhanced education by delineating how technological competencies translate into sustained learning behaviors through psychological mechanisms, thereby providing theoretically grounded and practically actionable insights into differentiated educational interventions.

## 2 Theories and assumptions

### 2.1 Artificial intelligence literacy and learning engagement

Artificial intelligence literacy refers to an individual’s comprehensive ability to understand, apply, and evaluate AI technologies and their societal implications, as well as to effectively interact with them (Kit et al., 2021). For university students, AI literacy serves as a foundational competency for effective learning, innovative practice, and responsible decision-making in the AI-driven era, directly affecting their future employability and lifelong learning capabilities (Laupichler et al., 2023). Learning engagement is defined as a persistent, positive psychological state related to learning, research, and career pursuits and is characterized by the core dimensions of vigor, dedication, and absorption (Schaufeli et al., 2002; Fang et al., 2008). It is a critical predictor of students’ academic achievement and plays a decisive role in enhancing their critical thinking and problem-solving abilities (Lei et al., 2018).

These theoretical propositions have received preliminary support from multiple empirical studies. At the level of direct antecedents of learning engagement, Guo X. et al. (2025) and Guo F. et al. (2025), based on a survey of over 70,000 undergraduates from 25 Chinese universities, found that the use of generative AI in academic tasks was positively associated with cognitive and emotional engagement. Nguyen et al. (2025) further demonstrated that the autonomy and interactivity of GenAI significantly enhance student engagement, with personalization positively moderating the relationship between autonomy and engagement. At the psychological mechanism level, Bai and Wang (2025) drawing on two-wave longitudinal data from 323 Chinese university students, revealed that GenAI interaction and output quality enhance learning outcomes by positively influencing learning motivation and creative self-efficacy, with learning motivation serving as a significant mediator between GenAI output quality and learning outcomes. Yin et al. (2025), using fsQCA, identified complementary effects between AI literacy and multidimensional learning engagement that synergistically promote academic performance. Furthermore, research grounded in the Technology Acceptance Model has demonstrated that intrinsic motivation, perceived usefulness, and perceived ease of use are significant predictors of students’ behavioral intention to adopt AI learning tools such as ChatGPT, and that AI tools, as user-friendly and engaging technologies, facilitate students’ active learning and learning engagement (Afzaal et al., 2025).

Despite these supportive findings, several critical limitations of the existing evidence warrant attention. The majority of the studies have focused on the effects of AI literacy on distal outcomes such as academic achievement (Wang, 2026) or have tested the reverse direction—i.e., the predictive role of learning engagement on AI literacy (Zhou and Peng, 2025). Direct examinations of the proximal relationship between AI literacy and learning engagement are scarce. Although some studies have addressed the relationship between AI use and learning engagement, their findings suggest potential nonlinear or conditional associations. For instance, Guo X. et al. (2025) and Guo F. et al. (2025) reported that, in the same study, generative AI use might actually reduce students’ active learning behaviors. This finding implies that the relationship between AI literacy and learning engagement may not simply be linear and positive; its specific manifestations in particular cultural and educational contexts or usage patterns require further investigation. Accordingly, this study proposes the following hypothesis:

*H1:* University students’ AI literacy has a significant positive predictive effect on their learning engagement.

### 2.2 The mediating effect of psychological capital

Although prior research has preliminarily established the positive effects of generative AI on learning engagement, as well as the direct association between AI literacy and learning engagement, the psychological mediating mechanisms and boundary conditions through which AI literacy translates into sustained learning engagement remain insufficiently examined. This research gap has been repeatedly highlighted in recent literature on human–AI collaboration and AI-supported learning (Zhou and Peng, 2025; Zhao, 2025). Within the broader framework of AI-supported learning, multiple lines of inquiry have begun to illuminate the potential psychological pathways underlying this relationship. Bai and Wang (2025), drawing on two-wave longitudinal survey data from 323 Chinese university students, found that GenAI interaction quality and output quality enhance learning outcomes by positively influencing learning motivation and creative self-efficacy. Complementing this line of inquiry, Chai (2026) demonstrated that AI literacy, in conjunction with expectancy-value beliefs and self-regulated learning, jointly shapes students’ learning engagement in AI-assisted learning environments. Collectively, these findings suggest that the effect of AI literacy on learning engagement is at least partially transmitted through psychological factors—specifically, learning motivation and creative self-efficacy have been preliminarily established as mediators, with self-regulated learning also playing a critical role.

Further extending this line of reasoning, systematic reviews have demonstrated that AI-supported self-directed learning positively affects learners’ needs for autonomy and competence and that learners’ prior knowledge serves as a facilitating factor for sustained use of AI resources (Younas et al., 2025). This finding suggests that the effect of AI literacy on learning engagement may operate through the satisfaction of basic psychological needs—a mechanism that lies at the core of psychological capital development. As a technological cognitive resource, AI literacy may facilitate the development of psychological capital through multiple pathways, including eliciting positive emotions, providing mastery experiences, and satisfying the needs for autonomy and competence (Fredrickson, 2001). Furthermore, in the context of AI and digital transformation, technological self-efficacy has been identified as a critical psychological factor influencing learners’ adaptation to AI-driven learning environments. However, empirical evidence indicates that the relationship between traditional technological self-efficacy and AI integration is not significant, suggesting that conventional teaching competencies may need to be enhanced through professional development to align with AI-driven innovations (Younas et al., 2026). This finding implies that the AI era requires a more comprehensive psychological resource construct that extends beyond traditional technological self-efficacy—a gap that psychological capital is well-positioned to fill.

Psychological capital (PsyCap) refers to an individual’s positive psychological state manifested during growth and development, which consists of four dimensions, namely self-efficacy, hope, optimism, and resilience (Luthans et al., 2007b). Shi et al. (2025) found that the level of artificial intelligence literacy positively predicts students’ writing performance. As a positive psychological resource for university students, PsyCap significantly enhances academic resilience, self-efficacy, and subjective well-being, serving as an important protective factor in coping with academic stress and promoting mental health (Luthans et al., 2007a, 2007b). According to the Broaden-and-Build Theory (Fredrickson, 2001), positive emotions broaden an individual’s thought–action repertoire and, in turn, build enduring personal resources. In AI-assisted learning contexts, the positive emotional experiences gained by students through the successful use of AI tools to complete learning tasks may gradually construct and accumulate psychological capital. As a technological cognitive resource, AI literacy may facilitate the development of psychological capital through multiple pathways, including eliciting positive emotions, providing mastery experiences, and satisfying basic psychological needs.

Empirical studies provide robust support for the proposed mediating role of psychological capital. First, direct evidence links AI engagement to psychological capital. Zhao (2025) found that interaction with AI as a digital companion significantly and positively predicted learners’ psychological capital among 550 online learners. Dou and Sun (2025), employing an experimental design grounded in self-determination theory, demonstrated that integrating AI tools significantly enhances English as a Foreign Language (EFL) students’ motivation, psychological well-being, and psychological capital. Second, evidence from related domains further corroborates this pathway. In the field of information literacy, Zhao (2024) found that information literacy self-efficacy mediated the relationship between information literacy and online learning engagement among 1,421 university students. Third, integrative frameworks have illuminated the psychological mechanism through which AI tools influence learning outcomes. Huang et al. (2026), drawing on the stimulus-organism-response framework and self-determination theory, revealed that GenAI tools satisfy students’ needs for autonomy and competence, stimulate positive psychological states, and thereby maintain creative confidence and sustained learning engagement. This mechanism is consistent with the Broaden-and-Build Theory (Fredrickson, 2001), which posits that positive emotions and need satisfaction contribute to the development of enduring psychological resources. Furthermore, a large body of research has consistently demonstrated that psychological capital significantly promotes learning engagement (Siu et al., 2014; Martínez et al., 2019; Liping and Yunfeng, 2023). Collectively, these lines of evidence—direct, indirect, and integrative—suggest that psychological capital serves as a key mediating mechanism linking AI literacy to learning engagement. Nevertheless, although prior studies have verified the associations between technological literacy, psychological resources, and learning behaviors from various perspectives, no study has directly tested the complete mediating pathway from AI literacy to learning engagement through psychological capital. Based on the above theoretical analysis and empirical evidence, this study proposes the following hypothesis:

*H2:* Psychological capital mediates the relationship between AI literacy and learning engagement.

### 2.3 The moderating effect of professional commitment

Professional commitment refers to the positive attitude and behavior through which university students identify with their chosen major and are willing to exert corresponding efforts. It comprises four dimensions: affective commitment, continuance commitment, normative commitment, and ideal commitment (Lian et al., 2005a, 2005b). For university students, professional commitment not only directly promotes learning engagement and academic achievement but also influences their career identity and the stability of their future professional development, serving as a key psychological foundation for high-quality talent cultivation (Yan and Long, 2008).

When university students develop a strong identification with their chosen major, their professional goals become internalized as personal goals, making them more inclined to invest their psychological resources in professional learning. In other words, for students with high professional commitment, the effect of psychological capital on promoting learning engagement is stronger. The expectancy-value theory (Eccles and Wigfield, 2002) further posits that an individual’s expectations and subjective task value jointly determine achievement behaviors. Students with high professional commitment attach greater value to professional learning; therefore, when they possess higher psychological capital, they are more willing to engage in professional learning. Conversely, even if low-commitment students have high psychological capital, their lack of value identification prevents them from translating it into action. From the perspective of identity maintenance, Self-Verification Theory (Swann, 1983) adds that highly committed students regard being an excellent learner in their major as part of their self-concept. When they possess high psychological capital, they actively seek to verify this identity through high levels of learning engagement, thereby strengthening the positive relationship between psychological capital and learning engagement.

Empirical studies have provided direct and indirect support for the positive moderating role of professional commitment. First, a meta-analysis (Han et al., 2025) that combined 68 experimental studies and 337 effect sizes found that, although the effect of generative AI on learning engagement was generally positive, there was significant heterogeneity, suggesting that its effectiveness highly depends on conditional factors such as subject background, knowledge type, and application method. The positive association between professional commitment and learning engagement is well established. Li (2025) found in a study of 846 teacher education students that professional commitment influenced learning engagement through chain mediation via self-control. Zhang et al. (2024) surveyed 1,009 nursing undergraduates and reported that the school education climate influenced professional commitment through the mediation of learning engagement, indicating a bidirectional positive relationship between the two. These findings provide a prerequisite for the role of professional commitment in strengthening the relationship between psychological resources and learning behaviors. Second, evidence for professional commitment as a moderator has begun to emerge. Wang and Zhang (2024), based on a survey of 1,032 university students, found that professional commitment partially mediated the relationship between psychological capital and learning engagement, and that this effect was moderated by gender, suggesting an interaction between professional commitment and psychological capital—specifically, that the predictive effect of psychological capital on learning engagement was stronger among students with high professional commitment. This finding provides direct support for the moderating effect hypothesized in this study. Dandan et al. (2025) studied 568 medical students and found that teacher leadership influenced academic burnout through the mediation of professional commitment, with professional commitment playing a protective role in reducing burnout, thereby confirming its positive moderating function. Third, from an intervention perspective, Liqi and Ping (2021) conducted a quasi-experimental study and found that a role-model education program significantly enhanced nursing students’ professional commitment, which in turn increased their learning engagement. This finding suggests that professional commitment is a malleable psychological variable, and its improvement helps amplify the facilitating effect of other positive factors on learning engagement.

Although previous studies have confirmed a positive relationship between professional commitment and learning engagement, as well as the moderating role between psychological resources and learning behaviors, whether professional commitment positively moderates the relationship between psychological capital and learning engagement within the specific mediating pathway from artificial intelligence literacy to learning engagement via psychological capital remains untested. In the context of Chinese higher education, university students face practical pressures related to major selection and transfer, resulting in considerable individual differences in professional commitment. Examining its moderating role therefore holds significant practical value for developing differentiated educational intervention strategies. Furthermore, AI has been shown to exert a double-edged effect on learning: while it can enhance learning motivation, creativity, and digital literacy, it may also induce negative outcomes, such as fear of missing out (FoMO), that impair academic performance (Khoso et al., 2025, 2026). The direction and magnitude of these effects depend on how individuals perceive and respond to AI technologies. This observation reinforces the necessity of examining professional commitment as a moderating variable; students with strong identification with their major may view AI as a tool for achieving professional goals, whereas those with low commitment may be more susceptible to AI-related anxiety.

Based on the above theoretical analysis and empirical evidence, this study proposes the following hypothesis:

*H3:* Professional commitment positively moderates the relationship between psychological capital and learning engagement.

### 2.4 Theoretical model

**Figure 1:**
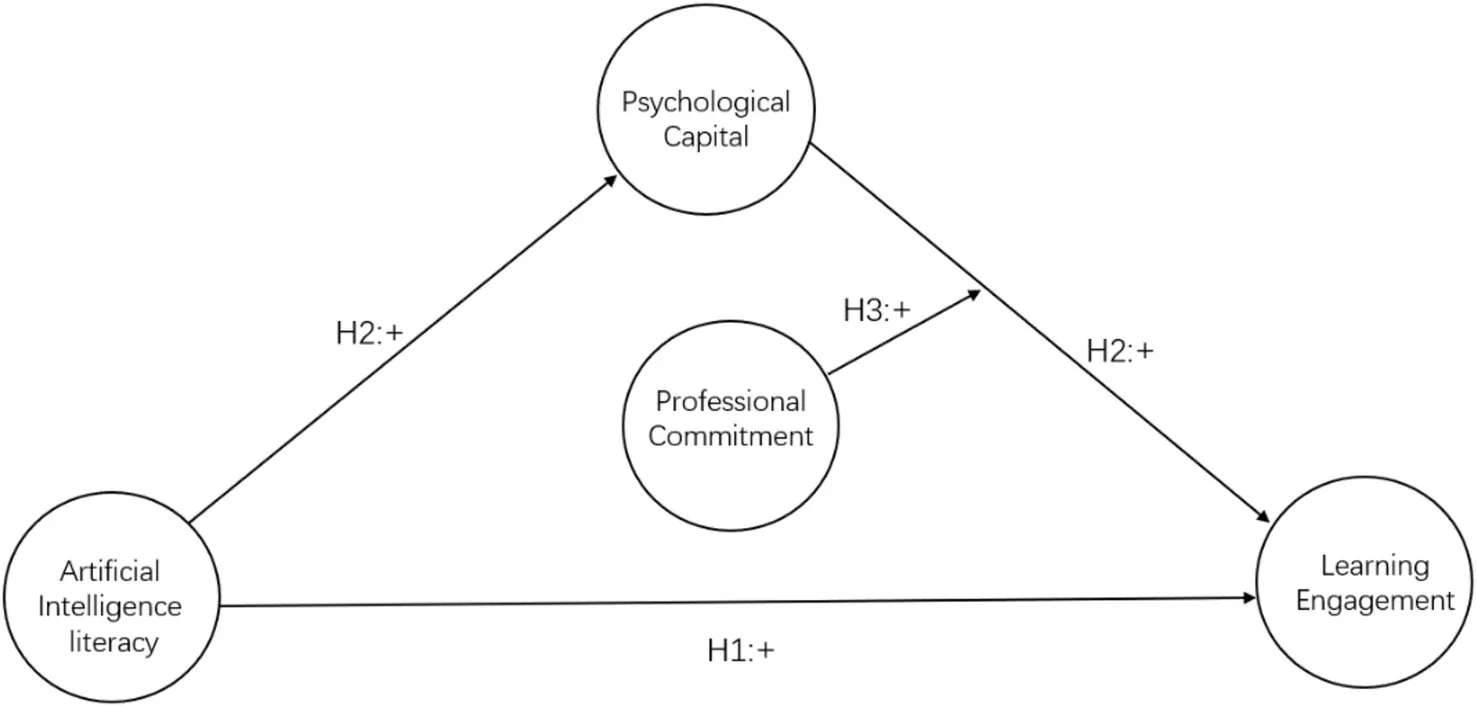
Hypothesized moderated mediation model. H1 to H3 denote hypotheses 1 to 3, and the symbols indicate the direction of the effects.

Based on the theoretical analysis and hypothesis development above, this study proposes a moderated mediation model. Specifically, Hypothesis H1 posits a direct positive predictive effect of artificial intelligence literacy on learning engagement, which serves as the baseline model of this study. Hypothesis H2 further proposes the mediating role of psychological capital in the relationship between AI literacy and learning engagement, such that AI literacy indirectly promotes learning engagement by enhancing university students’ psychological capital. Hypothesis H3 delineates the boundary condition of this mediating pathway, proposing that professional commitment positively moderates the relationship between psychological capital and learning engagement, thereby making the positive effect of psychological capital on learning engagement more pronounced among students with high professional commitment. Accordingly, this study puts forward a moderated mediation model (Figure 1).

## 3 Materials and methods

### 3.1 Participants

The survey was conducted among undergraduate students from four universities in Zhengzhou, Henan Province, between February and April 2026. To ensure a representative sample across key demographic characteristics, a stratified random sampling method was employed. The stratification variables were gender (male vs. female), grade level (freshman, sophomore, junior, and senior), and student leader status (holding an official leadership position in student organizations vs. not holding such a position). These variables were selected as stratification criteria based on prior empirical evidence demonstrating significant differences in AI literacy, learning engagement, psychological capital, and professional commitment across these demographic groups (Luo et al., 2008; Cui, 2012; Wang, 2013; Xu et al., 2014). The target population was first divided into mutually exclusive strata based on the full combination of the three stratification variables, yielding 16 strata (2 × 4 × 2). Within each stratum, participants were then randomly selected using a simple random sampling procedure. To maintain the representativeness of the sample, the sample size allocated to each stratum was proportional to the stratum’s representation in the overall population (proportional allocation). The survey was administered using a mixed-mode approach. A total of 719 questionnaires were distributed online via the Wenjuanxing platform, and 681 were distributed offline as paper questionnaires. The proportion of online versus offline distribution was determined according to each stratum’s proportion in the total sample, thereby ensuring that the mixed-mode administration did not introduce bias in the representation of different demographic groups. After excluding invalid responses (e.g., those with excessive missing data, patterned responding, or completion times below two standard deviations of the mean), 1,198 valid questionnaires were retained, yielding an effective response rate of 85.6% Jacob (n.d.).

Among the final analytical sample, 587 participants (49.0%) were female and 611 (51.0%) were male. Regarding grade level, 339 (28.3%) were freshmen, 291 (24.3%) were sophomores, 311 (26.0%) were juniors, and 257 (21.5%) were seniors. In addition, 237 participants (19.8%) reported holding a student leadership position.

To evaluate the representativeness of the sample, this study compared the demographic distribution of the sample with national benchmark data reported by the National Working Committee on Children and Women under the State Council of China (2025), which indicated that female students account for 50.76% of total higher education enrollment in China. The gender distribution of the present sample (49.0% female) closely matched this national figure. Furthermore, the proportions of student leaders (19.8%) and the grade-level distribution were consistent with the typical composition of Chinese university student populations as documented in national surveys (Ministry of Education of China, 2024). These comparisons collectively indicate that the sample structure adequately reflected the demographic characteristics of the broader undergraduate population and met the research requirements, thereby supporting the reliability and generalizability of the findings.

### 3.2 Instruments

The questionnaire used in this study consisted of two parts. The first part collected participants’ demographic information, including gender, grade level, and whether they served as student leaders. The second part comprised measures of the four focal variables: artificial intelligence literacy, professional commitment, psychological capital, and learning engagement.

Artificial intelligence literacy was assessed using the College Students’ Artificial Intelligence Literacy Scale developed by Kou (2025). This scale encompasses six dimensions—AI perception, AI comprehension, AI knowledge, AI skills, AI evaluation, and AI innovation—and includes 22 items. All items were rated on a 5-point Likert scale, with higher scores indicating higher levels of AI literacy. Professional commitment was measured using the Professional Commitment Scale developed by Lian et al. (2005a, 2005b). This scale comprises four dimensions: ideal commitment, affective commitment, continuance commitment, and normative commitment, with a total of 27 items. Among these, items 6, 8, and 12 were reverse-coded. The scale adopted a 5-point Likert scoring method, with higher scores reflecting higher levels of professional commitment. Psychological capital was assessed using the Positive Psychological Capital Questionnaire developed by Zhang et al. (2010). This questionnaire includes four dimensions—self-efficacy, resilience, hope, and optimism—and consists of 27 items. All items were rated on a 7-point Likert scale, with higher scores indicating higher levels of psychological capital. Learning engagement was measured using the Utrecht Work Engagement Scale for Students (UWES-S) developed by Schaufeli et al. (2002). This scale contains three dimensions (vigor, dedication, and absorption) and comprises 17 items, all rated on a 7-point Likert scale. Higher scores indicated higher levels of learning engagement.

Confirmatory factor analysis was conducted to evaluate the psychometric properties of the four scales. The Cronbach’s *α* coefficients for the four scales ranged from 0.93 to 0.99. The composite reliability (CR) coefficients ranged from 0.96 to 0.99. The average variance extracted (AVE) values ranged from 0.78 to 0.84. The factor loadings of all items ranged from 0.89 to 0.92.

### 3.3 Procedure

#### 3.3.1 Common method bias control

The study first employed Harman’s single-factor test to assess common method bias. To mitigate the risk of common method bias, several procedural remedies were implemented as recommended by Podsakoff et al. (2003). First, a temporal separation design was adopted by placing the predictor variables (AI literacy scale) before the outcome variables (learning engagement scale) in the questionnaire, thereby reducing respondents’ ability to infer the hypothesized relationships. Second, different scale anchors were employed for different constructs: AI literacy and professional commitment were measured using 5-point Likert scales, whereas psychological capital and learning engagement were measured using 7-point Likert scales, which helped to disrupt consistent response patterns across constructs. Third, we assured participants of the anonymity and confidentiality of their responses to minimize social desirability bias and encourage honest answering.

#### 3.3.2 Analytical strategy

Descriptive statistics and Pearson correlation analyses were conducted using SPSS 26.0 for the four core variables—artificial intelligence literacy, psychological capital, professional commitment, and learning engagement—to preliminarily examine the bivariate correlations among them. This study adopted partial least squares structural equation modeling (PLS-SEM) using SmartPLS 4.0 to test the proposed hypotheses. PLS-SEM was selected over covariance-based structural equation modeling (CB-SEM) for four reasons: First, this study is prediction-oriented, aiming to maximize the explained variance in learning engagement and identify key predictors, rather than testing a well-established theory with strong empirical support. Second, the proposed model involves considerable complexity, incorporating both mediation and moderation effects, a second-order construct, and an interaction term—all of which PLS-SEM handles effectively without the stringent identification constraints of CB-SEM. Third, PLS-SEM does not require multivariate normality assumptions and is robust to violations of distributional assumptions, making it suitable for educational and behavioral research data. Fourth, given the nascent stage of research on AI literacy in educational psychology, the theoretical framework remains exploratory, and PLS-SEM is recommended for such theory-development contexts (Henseler et al., 2015).

#### 3.3.3 Hypotheses testing

First, path analysis was performed to examine the direct predictive effect of AI literacy on learning engagement (Hypothesis H1). Next, the bootstrap method with 5,000 resampling iterations was used to test the mediating effect of psychological capital (Hypothesis H2), with a 95% bias-corrected confidence interval excluding zero as the criterion for statistical significance. Finally, to examine the moderating effect of professional commitment (Hypothesis H3), an interaction term (psychological capital × professional commitment) was constructed. The conditional indirect effect (i.e., moderated mediation) was estimated using the bootstrap method to test whether the mediating effect of psychological capital differed significantly across low, medium, and high levels of professional commitment. Simple slope analysis, conducted at one standard deviation above and below the mean of the moderator, was performed to reveal the specific direction and pattern of the moderating effect.

## 4 Results

### 4.1 Common method deviation test

The study employed Harman’s single-factor test by conducting an unrotated exploratory factor analysis of all measurement items. According to Podsakoff and Organ (1986), if the total variance explained by the first unrotated factor is below the 50% threshold, common method bias is not a serious concern. The results showed that multiple factors with eigenvalues greater than one were extracted, and the first factor accounted for 47.3% of the total variance, which is below the recommended threshold of 50%. Therefore, common method bias did not pose a serious problem in this study, and the data were deemed suitable for subsequent hypothesis testing.

### 4.2 Descriptive statistics

The study examined differences in artificial intelligence literacy, learning engagement, psychological capital, and professional commitment across gender, grade level, and student leader status using independent-samples *t*-tests and one-way analysis of variance (ANOVA). The independent-samples *t*-tests revealed that males scored significantly higher than females on artificial intelligence literacy (t = 5.51, *p* < 0.001). Females scored significantly higher than males on learning engagement (t = 9.74, *p* < 0.001). Student leaders reported significantly higher levels of learning engagement (t = 5.72, *p* < 0.001), psychological capital (t = 5.46, *p* < 0.001), and professional commitment (t = 5.79, *p* < 0.001) compared with non-leaders. The one-way ANOVA results showed significant grade differences in learning engagement (*F* = 8.19, *p* < 0.001), with freshmen and seniors exhibiting relatively higher levels. Significant grade differences were also found in professional commitment (*F* = 15.82, *p* < 0.001), with freshmen scoring relatively higher. These findings have been consistently supported by a large body of research (Luo et al., 2008; Cui, 2012; Wang, 2013; Xu et al., 2014; Zhang, 2020; Yang and Yang, 2022; Hu et al., 2023; Zhu et al., 2026).

### 4.3 Correlation analysis

#### 4.3.1 Bivariate correlation results

Pearson correlation analyses were conducted to examine the bivariate relationships among the core constructs. As presented in Table 1, AI literacy was positively correlated with learning engagement (r = 0.57, *p* < 0.01), and psychological capital was positively correlated with both AI literacy (r = 0.68, *p* < 0.01) and learning engagement (r = 0.65, *p* < 0.01). There was no significant relationship between professional commitment and artificial intelligence literacy (r = 0.001, *p* = 0.997) or learning engagement (r = 0.063, *p* = 0.063).

**Table 1:** Means, standard deviations, and intercorrelations for variables.

Variables	*M*	SD	AIL	LE	PSY	PC
AIL	3.13	1.29				
LE	4.62	0.79	0.57**			
PSY	4.94	1.07	0.68**	0.65**		
PC	3.46	1.23	0.001	0.063	0.03	

AIL = artificial intelligence literacy; LE = learning engagement; PSY = psychological capital; PC = professional commitment. *p < 0.05; **p < 0.01.

#### 4.3.2 Discriminant validity

To rigorously assess discriminant validity, the study employed the heterotrait–monotrait (HTMT) ratio of correlations (Henseler et al., 2015). As shown in the HTMT matrix, all HTMT values were well below the conservative threshold of 0.85. Specifically, the HTMT values for AI literacy and psychological capital (0.713), AI literacy with learning engagement (0.676), and psychological capital with learning engagement (0.718) all fell below the recommended cutoff, confirming that these constructs, while moderately associated, are empirically distinguishable.

#### 4.3.3 Multicollinearity assessment

To assess multicollinearity among the predictor variables, this study employed the variance inflation factor (VIF) test (Hair et al., 2011). The results showed that the VIF values for all predictor variables were below the conservative threshold of 5. The VIF for artificial intelligence literacy was 2.035, the VIF for psychological capital was 2.031, the VIF for professional commitment was 1.001, and the VIF for the interaction term (professional commitment × psychological capital) was 1.004. These results indicate that there is no multicollinearity problem in this study and that the predictor variables maintain sufficient independence, ensuring the stability and reliability of subsequent path coefficient estimations.

### 4.4 Effect of artificial intelligence literacy on learning engagement

This study constructed a model examining the effect of artificial intelligence literacy on learning engagement and analyzed it using partial least squares structural equation modeling (PLS-SEM). The results showed that the model explained a substantial proportion of the variance, with a coefficient of determination (*R*^2^ = 0.446), indicating adequate explanatory power. The standardized path coefficient from artificial intelligence literacy to learning engagement was *β* = 0.668 (*p* < 0.001), demonstrating that this effect was statistically significant. Thus, Hypothesis 1 was supported.

### 4.5 Moderating model checking

To test the mediating role of psychological capital between artificial intelligence literacy and learning engagement, the study constructed a path model in which AI literacy influenced learning engagement via psychological capital. The results showed that the coefficient of determination (*R*^2^) was 0.507 for psychological capital and 0.497 for learning engagement, indicating adequate explanatory power.

**Figure 2:**
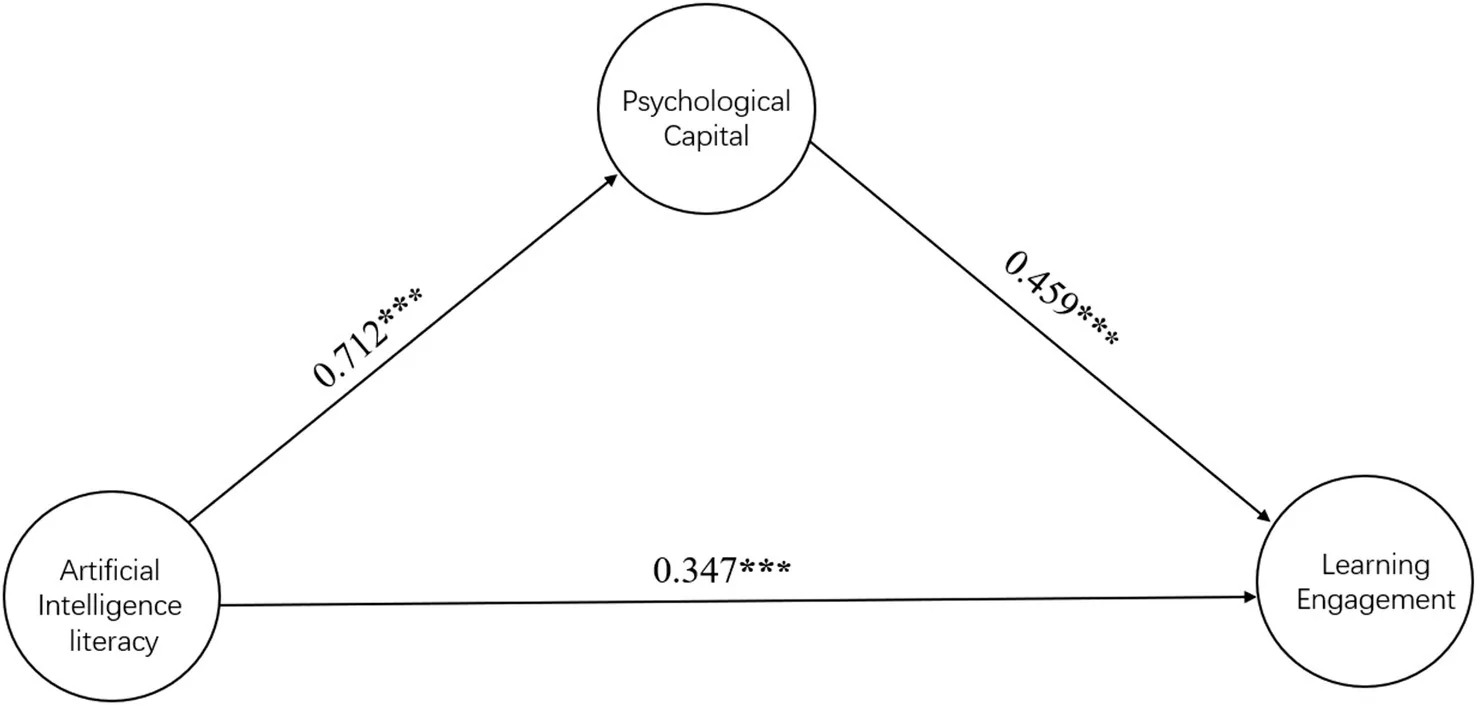
The mediating role of psychological capital in the relationship between artificial intelligence literacy and learning engagement. ***p < 0 0.001.

In this model (Figure 2), the standardized path coefficient from AI literacy to psychological capital was significant (*β* = 0.712, *p* < 0.001), and the standardized path coefficient from psychological capital to learning engagement was also significant (β = 0.459, *p* < 0.001). After including psychological capital in the model, the standardized path coefficient from AI literacy to learning engagement remained significant (β = 0.347, *p* < 0.001).

The bootstrap procedure implemented in SmartPLS 4.0 was employed, with 5,000 resampling iterations performed on the original data, and 95% confidence intervals were estimated. A mediation effect was considered statistically significant if the confidence interval excluded zero. As shown in Table 2, the indirect effect was 0.327, with a 95% confidence interval of [0.289, 0.365], which excluded zero. These results indicate that psychological capital mediates the relationship between artificial intelligence literacy and learning engagement. Effect size analysis revealed that the variance accounted for (VAF) by mediation was 48.5%, representing partial mediation with meaningful practical significance (Hair et al., 2017). Thus, Hypothesis 2 was supported.

**Table 2:** Bootstrap analysis of the mediating effect of psychological capital on the relationship between artificial intelligence literacy and learning engagement.

Mediation path	Effect estimate	95% confidence intervals			
		Percentile		Bias-corrected	
		Lower bound	Upper bound	Lower bound	Upper bound
AIL→PSY → LE	0.327	0.289	0.365	0.289	0.365

AIL = artificial intelligence literacy; LE = learning engagement; PSY = psychological capital.

### 4.6 Mediating model checking

#### 4.6.1 Significance test of interaction terms

**Figure 3:**
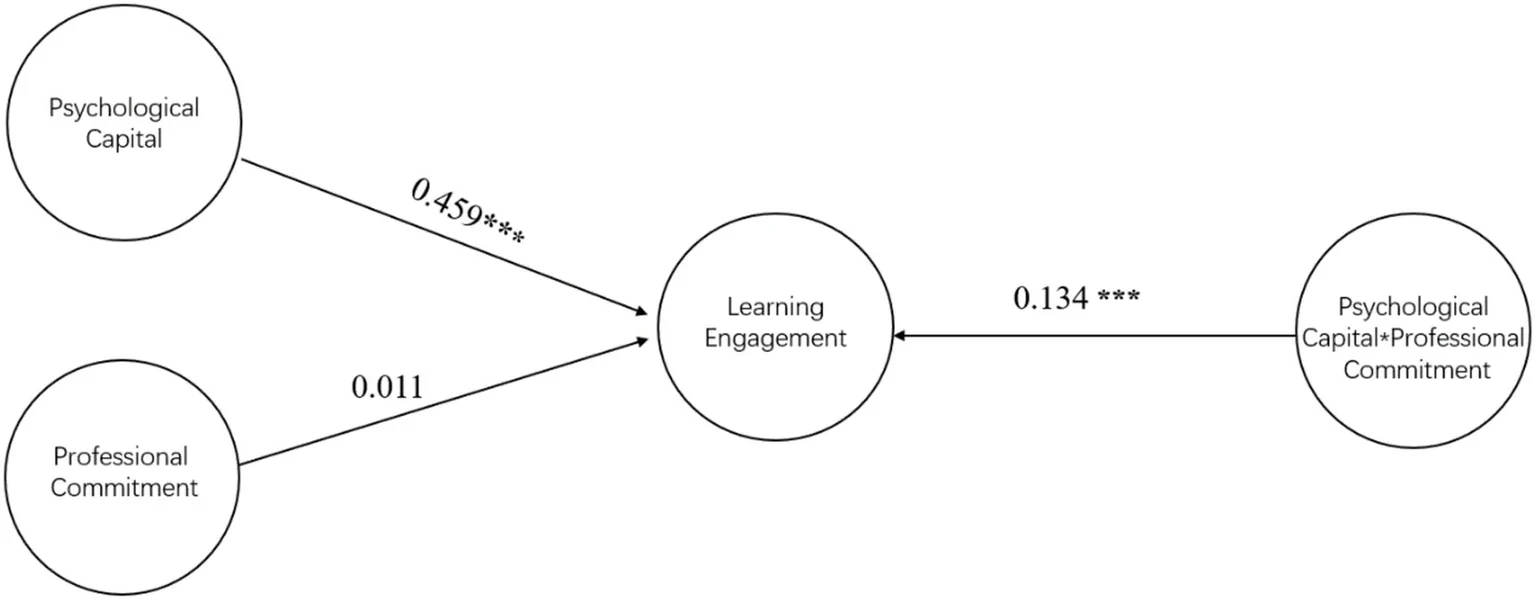
Moderating effect of professional commitment on the relationship between psychological capital and learning engagement. ***p < 0.001.

To test the moderating role of professional commitment in the relationship between psychological capital and learning engagement, an interaction term (psychological capital × professional commitment, abbreviated as PSY × PC) was constructed. Path analysis was conducted using partial least squares structural equation modeling (PLS-SEM), and the results are presented in Figure 3. The main effect of psychological capital on learning engagement was significant (*β* = 0.459, t = 17.380, *p* < 0.001). The main effect of professional commitment on learning engagement was not significant (β = 0.011, t = 0.384, *p* = 0.701). Importantly, the path coefficient of the interaction term (PSY × PC) on learning engagement was significant (*β* = 0.134, t = 5.288, *p* < 0.001). These findings indicate that professional commitment positively moderates the relationship between psychological capital and learning engagement.

#### 4.6.2 Moderating effect of professional commitment

**Figure 4:**
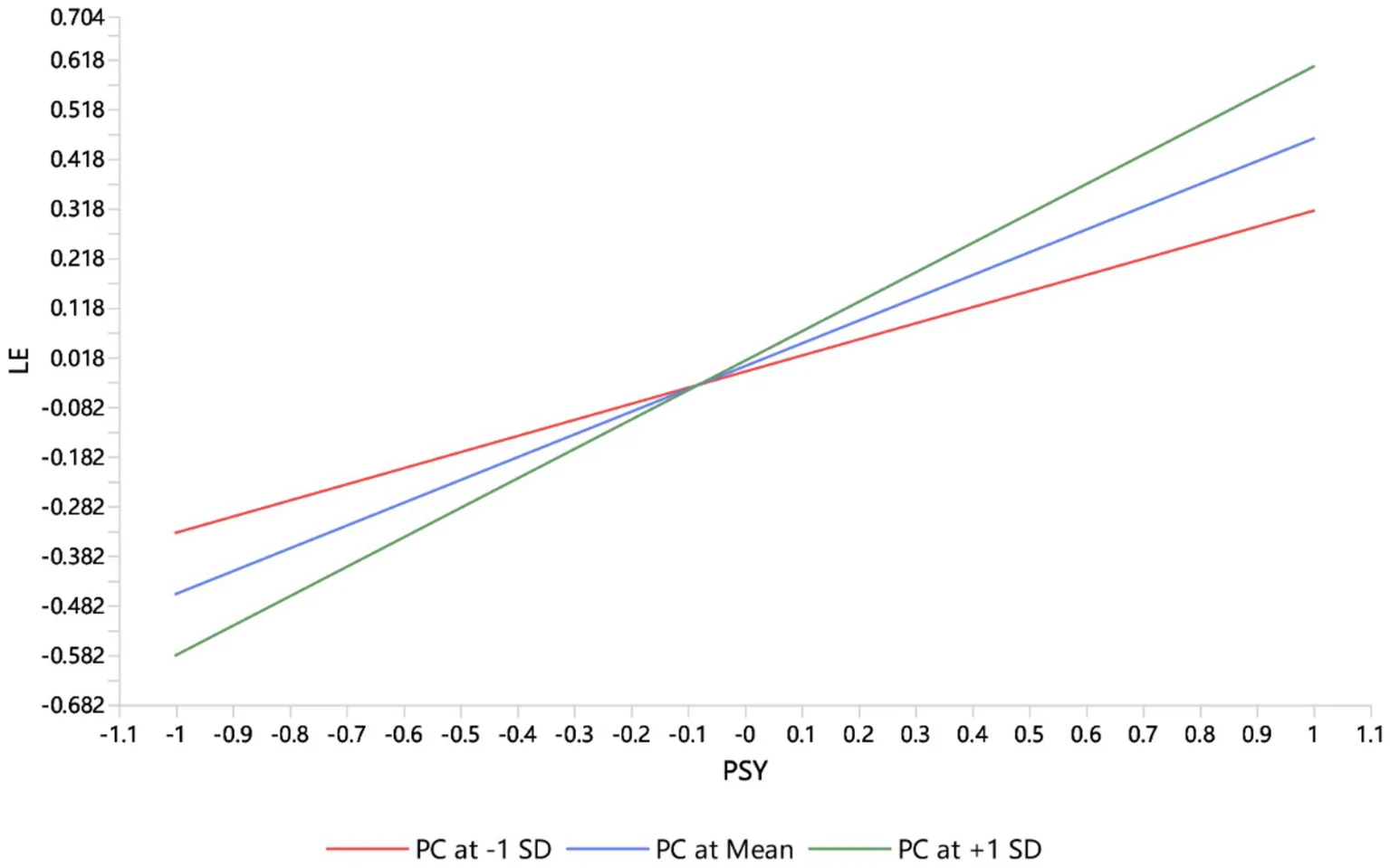
Simple slope plot illustrating the moderating effect of professional commitment. LE = learning engagement; PSY = psychological capital; PC = professional commitment.

To further examine the moderating role of professional commitment in the relationship between psychological capital and learning engagement, simple slope analyses were conducted by dividing professional commitment into high and low groups at one standard deviation above and below the mean, respectively (Figure 4). The results showed that the slope of psychological capital predicting learning engagement was 0.324 in the low professional commitment group (i.e., one standard deviation below the mean), 0.459 in the moderate professional commitment group (i.e., at the mean level), and increased to 0.593 in the high professional commitment group (i.e., one standard deviation above the mean). These slope values indicate that the positive effect of psychological capital on learning engagement becomes progressively stronger as the level of professional commitment increases. Thus, these findings confirm Hypothesis H3.

#### 4.6.3 Moderated mediation effects

Table 3 presents the conditional indirect effects estimated using the bootstrap method with 5,000 resamples. The results showed that the indirect effect of AI literacy on learning engagement via psychological capital was significant across different levels of professional commitment. Specifically, the indirect effect was 0.204 (SE = 0.023, 95% CI [0.160, 0.249], *p* < 0.001) for low professional commitment (one standard deviation below the mean), 0.289 (SE = 0.019, 95% CI [0.253, 0.326], *p* < 0.001) for the mean level of professional commitment, and 0.374 (SE = 0.022, 95% CI [0.332, 0.418], *p* < 0.001) for high professional commitment (one standard deviation above the mean). Furthermore, the index of moderated mediation was significant (*β* = 0.307, SE = 0.024, 95% CI [0.099, 0.176], *p* < 0.001). Collectively, these findings indicate that professional commitment significantly moderates the indirect effect of AI literacy on learning engagement via psychological capital and that this indirect effect increases progressively as the level of professional commitment rises.

**Table 3:** Moderated mediation.

Relationships	*β*	SE	T	2.5%	97.5%
AIL→ PSY → LE conditional on PC + 1 SD	0.374^⁎⁎⁎^	0.022	16.84	0.332	0.418
AIL→ PSY → LE conditional on PC -1 SD	0.204^⁎⁎⁎^	0.023	8.798	0.16	0.249
AIL→ PSY → LE conditional on PC Mean	0.289^⁎⁎⁎^	0.019	15.21	0.253	0.326
Index of conditional mediation	0.307^⁎⁎⁎^	0.024	12.799	0.099	0.176

AIL, artificial intelligence literacy; LE, learning engagement; PSY, psychological capital; PC, professional commitment. ***p < 0.001.

#### 4.6.4 Effect size interpretation

Although the moderating effect observed in this study was statistically significant (β = 0.134, *p* < 0.001, f^2^ = 0.018), its effect size fell within the small-to-medium range. However, from the perspective of educational intervention, this seemingly limited effect size has significant practical implications. Specifically, in students with a higher level of professional commitment, the predictive slope of psychological capital on learning engagement was β = 0.593, whereas in students with low professional commitment, it was only β = 0.324, meaning that the former was 83.0% higher than the latter. In other words, raising students’ professional commitment from a low to a high level can nearly double the efficiency of psychological capital in promoting learning engagement. Furthermore, the conditional indirect effect increased from 0.204 (95% CI [0.160, 0.249]) in the low professional commitment group to 0.374 (95% CI [0.332, 0.418]) in the high professional commitment group, an increase of 83.3%. This monotonic increasing pattern indicates that professional commitment not only acts as a boundary condition for the functioning of psychological capital but also plays a quantifiable “context-amplifier” role. That is, for every unit increase in professional commitment level, the indirect effect of AI literacy on learning engagement through psychological capital significantly strengthens. These findings provide clear quantitative evidence that universities can implement differentiated educational intervention strategies.

## 5 Discussion

### 5.1 Predictive effect of artificial intelligence literacy on learning engagement

This study found that artificial intelligence literacy had a significant and direct positive effect on learning engagement.

This finding is consistent with the core tenets of Social Cognitive Theory (Bandura, 2001). This theory posits that personal cognitive resources are key antecedents driving behavior. In the context of artificial intelligence being deeply embedded in learning environments, AI literacy among university students can be regarded as an important personal cognitive resource. Students with higher AI literacy are better able to utilize intelligent tools for information retrieval, knowledge construction, and problem-solving, thereby demonstrating higher levels of vigor, dedication, and absorption in technology-intensive learning contexts. In other words, AI literacy directly promotes learning engagement by enhancing individuals’ cognitive operational efficiency.

Conservation of Resources Theory (Hobfoll, 2002) offers a complementary explanation from the perspective of resource acquisition and preservation. This theory suggests that individuals strive to acquire, preserve, and enhance resources they value and that the investment of initial resources generates a gain spiral effect. As a valuable technological cognitive resource, AI literacy helps students reduce cognitive load and improve academic self-efficacy during the learning process, thereby mitigating the depletion of psychological resources and fostering active and sustained learning engagement. When students complete learning tasks with a high level of AI literacy, such successful experiences further strengthen their resource reserves, forming a positive feedback loop.

Cognitive load theory (Kalyuga, 2011) posits that cognitive load can be classified into intrinsic and extraneous load, where intrinsic load is determined by the complexity of the learning task, whereas extraneous load is associated with environmental factors. Li et al. (2026) noted that the integration of artificial intelligence in higher education brings challenges related to technological adaptation and cognitive load, implying that whether students can effectively leverage AI to reduce cognitive load and thereby enhance learning engagement depends largely on their level of AI literacy.

This result echoes the conclusion of Guo X. et al. (2025) and Guo F. et al. (2025), based on a survey of undergraduate students from 25 Chinese universities, that the use of generative AI in academic tasks is positively associated with cognitive and emotional engagement. This result also aligns with the meta-analytic evidence provided by Younas et al. (2025), which demonstrated that AI technologies—such as intelligent tutoring systems and adaptive learning platforms—significantly improve personalized learning and learning engagement. Moreover, this finding is consistent with meta-analytic conclusions in the AI-supported learning literature: Wang and Guo (2025), synthesizing 31 empirical studies (91 effect sizes), confirmed that GenAI exerts a significant positive effect on overall learning engagement, with the strongest effect observed on cognitive engagement. Complementing this evidence from the motivational pathway, Bai and Wang (2025) found that GenAI interaction quality enhances learning outcomes by positively influencing learning motivation and creative self-efficacy. Ren et al. (2025) found that digital competence, via self‑efficacy, significantly predicts perceived interactivity in AI‑supported learning contexts. Chai (2026), based on expectancy‑value theory, confirmed that AI usage, motivation, and self‑efficacy jointly predict learning engagement.

### 5.2 The mediating mechanism of psychological capital

This study further revealed the mediating role of psychological capital in the relationship between artificial intelligence literacy and learning engagement.

Self-Determination Theory (Ryan and Deci, 2000) offers a complementary explanation for the transformation of AI literacy into psychological capital. This theory posits that the satisfaction of three basic psychological needs—autonomy, competence, and relatedness—is a prerequisite for the generation of intrinsic motivation. In AI-assisted learning contexts, students with higher AI literacy are able to autonomously select and use intelligent tools and solve learning problems in creative ways. This process simultaneously satisfies the needs for autonomy and competence. The satisfaction of these basic psychological needs subsequently activates intrinsic motivation and facilitates the accumulation of psychological resources. Unlike previous studies that merely described the mediating pathway within a technology-enhanced learning framework, this study explicitly positions the satisfaction of basic psychological needs as the key psychological mechanism through which technological literacy is converted into psychological capital, thereby deepening understanding of this mediating process.

Furthermore, the Broaden-and-Build Theory (Fredrickson, 2001) provides an affective explanation for the above mechanism. This theory proposes that positive emotions broaden an individual’s momentary thought–action repertoires and, over time, build enduring personal resources. In the process of AI-assisted learning, the sense of achievement and satisfaction that students gain from successfully using intelligent tools to complete tasks represent typical positive emotional experiences. Through repeated occurrence, these transient emotions become consolidated, eventually forming relatively stable self-efficacy and an optimistic orientation—core components of psychological capital. This emotional accumulation pathway has rarely been systematically examined in educational literature. The findings of this study extend the understanding of the psychological effects of AI literacy by highlighting the mediating role of emotional experience in the transformation of technological resources into psychological resources. Once psychological capital is formed, its four dimensions—self-efficacy, hope, optimism, and resilience—synergistically drive learning engagement. Self-efficacy enables students to believe in their ability to meet learning challenges; hope guides them to set and pursue academic goals; optimism helps them positively confront difficulties; and resilience allows them to recover quickly from setbacks. Together, these psychological resources prompt students to demonstrate higher levels of vigor, dedication, and absorption in learning activities. The findings suggest that enhancing the educational benefits necessitates not only technical skills but also systematic cultivation of psychological capital by satisfying basic needs and eliciting positive emotions.

These findings are highly consistent with those of Huang and Liang (2026) in AI-supported learning contexts, who demonstrated that psychological capital and emotion regulation jointly predict learning engagement, underscoring the pivotal role of psychological resources in AI-supported learning environments. Furthermore, human–AI collaboration theory provides a contextualized explanation for this transformation process. In the process of human–AI co-creation, the quality of AI interaction positively predicts individuals’ cognitive engagement (Pramod, 2026), which, as a positive psychological state, constitutes a key manifestation of psychological capital. Building upon this evidence, this study further extends this pathway backward to encompass the full chain from AI literacy to psychological capital and then to learning engagement, thereby offering a more comprehensive theoretical account of how technological resources are transformed into sustained learning behaviors through psychological capital.

### 5.3 The positive moderating effect of professional commitment

According to Social Identity Theory (Tajfel and Turner, 1979), individuals derive their self-concept and self-esteem by belonging to specific social groups, and such group identification significantly shapes their attitudes and behaviors. When university students develop a strong identification with their chosen major, membership in that professional group becomes a highly salient and emotionally attached component of their self-concept, and professional goals become internalized as personal goals. Driven by this identity, individuals tend to align their behaviors with the values and normative expectations of the group, thereby engaging more actively in identity-consistent learning behaviors when their psychological capital is enhanced. When psychological capital activates learning motivation through the self-efficacy belief that one can successfully complete learning tasks, this motivation is more readily channeled into a behavioral trajectory consistent with professional identity among students with high levels of professional commitment. Conversely, for students with low professional commitment, whose identity is relatively ambiguous, the learning motivation elicited by psychological capital lacks a clear identity carrier and, thus, is difficult to translate effectively into action.

Self-Verification Theory (Swann, 1983) posits that individuals have a fundamental motive to seek consistency between others’ perceptions of them and their own self-perceptions, and they actively engage in behaviors to verify and reinforce their self-concept. Students with high professional commitment position themselves as excellent learners in their major and incorporate this identity into the core of their self-concept. When their psychological capital increases, their academic self-efficacy, optimistic orientation, and other psychological resources are strengthened; concurrently, the identity expectation of being a proactive and competent learner in their major is also activated. At this point, students need to verify and confirm this self-concept through high levels of actual learning behavior. If an increase in psychological capital is not accompanied by corresponding learning engagement, they face cognitive dissonance (Festinger, 1957) between their self-conception and their actual behavior. Driven by this psychological tension, individuals develop a strong intrinsic motivation to adjust their behavior to close the gap, namely, to verify and reinforce their professional identity through high learning engagement. This identity-verification motive further strengthens the positive transformation of psychological capital into learning engagement.

This theoretical interpretation is further supported by an unanticipated pattern in the correlation matrix: professional commitment exhibited near-zero correlations with both AI literacy (r = 0.001) and learning engagement (r = 0.063). This finding, while initially unexpected, supports rather than undermines the theoretical rationale for treating professional commitment as a moderator. Had professional commitment been strongly correlated with either AI literacy or learning engagement, it would have been difficult to disentangle its moderating role from its direct effects. Its empirical independence from both the predictor and the outcome variable allows professional commitment to offer unique explanatory power by conditioning the strength of the psychological capital–learning engagement relationship without being confounded by direct associations with the predictor or the outcome. In other words, professional commitment functions as a contextual amplifier—it does not itself drive engagement but rather strengthens the extent to which psychological capital translates into engagement. This pattern reduces the likelihood of the alternative explanation that professional commitment merely acts as a proxy for AI literacy and learning engagement, thereby reinforcing the validity of its moderating role in the present model.

The moderating effect identified in this study is both related to and distinct from existing findings on professional commitment and career identity in the field of educational psychology. First, several studies have confirmed the direct positive predictive effect of professional commitment on learning engagement (Zhang et al., 2024), but its role as a moderator has been less frequently examined. Second, Wang and Zhang (2024) found that professional commitment partially mediated the relationship between psychological capital and learning engagement, and this effect was moderated by gender, suggesting an interactive relationship between professional commitment and psychological capital. By positioning professional commitment as a moderator, this study explicitly delineates the boundary condition under which psychological capital affects learning engagement, thereby complementing the above findings. Furthermore, Dandan et al. (2025) found that teacher leadership influenced academic burnout through the mediation of professional commitment, with professional commitment playing a protective role, indirectly supporting its positive moderating function. The findings of this study further indicate that professional commitment not only moderates the influence of external factors but also amplifies the effect of internal psychological resources. It is noteworthy that AI literacy is, in essence, a comprehensive competence demonstrated by individuals during human–AI interaction, and human–AI collaboration constitutes the core context in which AI literacy operates (Sidra and Mason, 2026). Consequently, understanding how AI literacy influences learning engagement through psychological capital necessitates a thorough consideration of human–AI collaboration as a contextual factor, which in turn provides a broader theoretical foundation for incorporating professional commitment as a moderating variable into the present model. For students with high professional commitment, the positive experiences gained from human–AI collaboration directly validate their identity as professional learners. To further verify and reinforce this identity, they are more inclined to invest their psychological resources in professional learning. In contrast, even if students with low professional commitment obtain favorable experiences from human–AI collaboration, these experiences are unlikely to translate into sustained professional learning engagement due to the lack of professional goal orientation that could channel such experiences into meaningful action.

### 5.4 Theoretical contributions

This study makes several interconnected theoretical contributions to the educational psychology and AI literacy literature.

The primary theoretical contribution of this study lies in establishing Conservation of Resources Theory as the core theoretical foundation, thereby constructing a multi-theoretical integrative explanatory framework. COR theory, with its resource gain spiral as the central explanatory thread, unifies the complete transformation pathway—from AI literacy (technological cognitive resources) to psychological capital (psychological resources) to learning engagement (behavioral resources)—within a single theoretical model. Complementing this core framework, the supplementary theoretical perspectives serve to elucidate the underlying mechanisms at multiple levels: Social Cognitive Theory accounts for the cognitive mechanism, Broaden-and-Build Theory explains the affective mechanism, Self-Determination Theory addresses the motivational mechanism, and Social Identity Theory together with Self-Verification Theory illuminate the identity-based mechanism. Collectively, these theoretical integrations provide a comprehensive, multi-layered account of how AI literacy translates into sustained learning engagement.

Second, prior research on AI-supported learning has predominantly examined isolated psychological factors such as self-efficacy and learning motivation; this study introduces psychological capital as a comprehensive positive psychological resource construct encompassing four dimensions—self-efficacy, hope, optimism, and resilience. In doing so, it reveals the composite mechanism through which AI literacy drives learning engagement via the synergistic effects of multidimensional psychological resources. This theoretical extension moves beyond the prevailing analytical paradigm of single-factor mediation that has characterized previous investigations in this domain.

Third, by introducing psychological capital as a comprehensive construct into AI literacy research, this study transcends prior investigations that have focused primarily on singular psychological factors such as technological self-efficacy (Younas et al., 2026). Systematic reviews have demonstrated that AI-supported self-directed learning promotes learning by satisfying needs for autonomy and competence (Younas et al., 2025). This study extends this pathway further backward, revealing the complete chain from AI literacy to psychological capital to learning engagement, thereby elucidating the transformative mechanism from technological cognitive resources to psychological resources and ultimately to behavioral resources.

Fourth, this study identifies and empirically validates professional commitment as a pivotal boundary condition that moderates the relationship between psychological capital and learning engagement. Although previous studies have confirmed the positive association between professional commitment and learning engagement (Zhang et al., 2024; Li, 2025), the moderating role of professional commitment in the psychological capital–learning engagement linkage has remained theoretically underspecified and empirically untested within the AI literacy framework. The present findings reveal that professional commitment functions as an “identity activator” that channels psychological resources into identity-consistent learning behaviors. By repositioning professional commitment from a direct predictor to a contextual amplifier, this study extends the theoretical scope of professional commitment research and offers a more nuanced understanding of when and for whom psychological capital has the greatest effect.

Fifth, by integrating AI literacy, psychological capital, and professional commitment into a unified moderated mediation framework, this study provides a comprehensive theoretical account of how technological competencies translate into sustained learning engagement. The finding that the indirect effect of AI literacy on learning engagement via psychological capital progressively increases as professional commitment increases not only offers a unifying theoretical perspective that reconciles previously fragmented lines of inquiry—namely, the AI literacy–engagement, psychological capital–engagement, and commitment–engagement relationships—but also provides a parsimonious explanation for the previously observed inconsistent findings regarding the effects of AI use on learning behaviors (Guo X. et al., 2025; Guo F. et al., 2025). These results suggest that the educational benefits of AI literacy are not automatic; rather, their realization depends on the simultaneous presence of psychological resources and professional identity. This integrated perspective moves the field beyond simplistic main-effect models toward a more context-sensitive understanding of AI’s role in higher education, thereby setting a new agenda for future research on technology-enhanced learning and psychological resource mobilization.

From the perspective of digital readiness, the findings of this study further elucidate the pivotal role of psychological readiness in digital learning. Research has demonstrated that psychological factors—including interest, attitude, and self-efficacy—constitute significant antecedents of AI literacy (Malik et al., 2026). Complementing this line of inquiry, this study reveals that AI literacy, in turn, contributes to the development of psychological capital, which subsequently drives learning engagement. Collectively, these two lines of evidence delineate a broader pathway linking psychological readiness to AI literacy, psychological capital, and ultimately, learning engagement. Thus, this study advances digital readiness research from static description to dynamic mechanism inquiry, thereby achieving a substantial theoretical upgrade in this evolving field.

### 5.5 Implications for educational practice

The findings of this study offer several actionable implications for higher education institutions seeking to leverage AI literacy for enhanced learning engagement.

First, psychological capital serves as a critical transmission channel for translating AI literacy into learning engagement. Universities should recognize that AI skills training alone is insufficient. To maximize the educational return on their AI literacy investments, institutions must simultaneously cultivate students’ psychological resources. This can be achieved by designing learning activities that generate success experiences and positive feedback—such as scaffolded AI-assisted projects with progressive difficulty levels—which systematically build students’ self-efficacy, hope, optimism, and resilience (Burke and Cooper, 2008). In practice, this means integrating psychological capital development into the core curriculum rather than treating it as an ancillary student service.

Second, professional commitment functions as a contextual amplifier that substantially increases the extent to which psychological capital translates into learning engagement. Students with strong professional commitment derive significantly greater engagement benefits from their psychological resources than those with low professional commitment. This finding carries a clear practical directive: for students with weak professional identity—particularly freshmen, transfer students, or those who entered their major through involuntary assignment—institutions should prioritize identity-building interventions before investing heavily in skill training or psychological capital development. Effective strategies include professional orientation courses, alumni career storytelling, industry-university-research practice programs, and faculty mentoring that explicitly connects academic content to career relevance (Li et al., 2023). Chunqing et al. (2023), in a randomized experiment with 36,482 medical students, found that a role model‑based information intervention significantly reduced students’ dropout intention by 2.7 percentage points, indicating that role modeling is an effective strategy for enhancing professional commitment. Without such foundational identity work, even students with adequate psychological resources may fail to convert these resources into sustained learning behaviors.

Third, differentiated intervention strategies should be tailored to students’ profiles. Students with high AI literacy but low psychological capital benefit most from small-step, achievable group projects that enable gradual accumulation of success experiences (Guo X. et al., 2025; Guo F. et al., 2025). Students with high psychological capital but low professional commitment require identity-focused interventions before skill training can be effective (Tal-Saban et al., 2024). Students with both low AI literacy and low psychological capital need foundational support addressing both dimensions simultaneously.

Fourth, universities should incorporate psychological capital and professional commitment into their academic monitoring systems. Regular screening can identify two priority groups—high AI literacy/low psychological capital and high psychological capital/low professional commitment—enabling targeted, cost-effective interventions that prevent the misallocation of educational resources (Li, 2025). This monitoring approach also provides a data-driven basis for evaluating the effectiveness of institutional interventions over time.

In sum, this study demonstrates that AI literacy alone is insufficient to guarantee learning engagement. Its educational benefits depend critically on a two-step psychological process: the accumulation of psychological capital and its activation through professional commitment. Higher education institutions should therefore adopt an integrated approach that simultaneously addresses technical skills, psychological resources, and professional identity to maximize the educational return on AI integration.

### 5.6 Limitations and future directions

This study has several limitations. First, the cross-sectional design does not allow for strict causal inferences regarding the directional relationships among the variables. Although the hypothesized model is theoretically grounded and empirically supported by the data, alternative causal directions cannot be entirely ruled out. For instance, it is plausible that students with higher learning engagement may be more motivated to develop their AI literacy or that reciprocal relationships exist among the constructs. Future research should adopt longitudinal designs or experimental interventions—such as AI literacy training programs with pretest–posttest assessments—to further examine causal directions and provide stronger evidence for the temporal precedence of AI literacy over psychological capital and learning engagement.

Second, the sample was limited to undergraduate students from four universities in Zhengzhou, which may restrict the generalizability of the findings. Subsequent studies could test the robustness of the proposed model across different regions, academic disciplines, and educational levels.

Third, this study only examined the mediating role of psychological capital as a composite construct. Future research could explore more specific mediators, such as AI self-efficacy and technological resilience, to disentangle the distinct effects of each component of psychological resources.

Finally, with the rapid iteration of generative artificial intelligence technologies, how university students use AI is shifting from low-frequency, shallow instrumental applications toward high-frequency, deep collaborative co-creation. This evolution implies that the conceptualization and measurement of AI literacy need to evolve accordingly. Traditional literacy assessments have largely focused on operational skills and basic knowledge, whereas future evaluation systems should pay greater attention to students’ adaptability and reflective abilities across different usage patterns. Subsequent research could develop assessment tools capable of dynamically capturing these differences, thereby enabling a more precise reflection of the structural evolution of AI literacy and its effects on learning engagement.

## 6 Conclusion

This study constructed and empirically tested a moderated mediation model to examine how artificial intelligence literacy influences university students’ learning engagement. The results revealed three main findings. First, AI literacy directly and positively predicted learning engagement. Second, psychological capital partially mediated this relationship. Third, professional commitment positively moderated the relationship between psychological capital and learning engagement, such that the indirect effect of AI literacy on learning engagement via psychological capital increased progressively as levels of professional commitment increased. These findings delineate a resource transformation pathway through which cognitive resources are transformed into psychological resources and subsequently into behavioral resources, while identifying professional commitment as a critical boundary condition. From a practical perspective, higher education institutions should integrate psychological capital cultivation and professional identity strengthening into AI literacy training to maximize the educational benefits of AI technologies and convert students’ technical competencies into sustained engagement in learning.

## Data Availability

The raw data supporting the conclusions of this article will be made available by the authors, without undue reservation.
